# Associations of age and sexual abstinence duration with semen parameters in normozoospermic men

**DOI:** 10.1590/1806-9282.20260425

**Published:** 2026-10-12

**Authors:** Elif Seda Özdemir, Abuzer Öztürk

**Affiliations:** 1 Sivas Numune Hospital, Andrology Laboratory – Sivas, Turkey.; 2 Sivas Numune Hospital, Department of Urology – Sivas, Turkey.

**Keywords:** Semen analysis, Sperm motility, Age factors, Sexual abstinence

## Abstract

**OBJECTIVE:**

The aim of this study was to evaluate the effects of age and duration of sexual abstinence on semen parameters in normozoospermic men and to explore their relative contributions within physiologically normal ranges.

**METHODS:**

This retrospective study included 412 normozoospermic men defined according to World Health Organization 2010 criteria. Semen volume, sperm concentration, and motility parameters were analyzed. Participants were stratified by age and abstinence duration. Correlation analyses and multivariate linear regression models were performed.

**RESULTS:**

Increasing age was modestly associated with lower total and progressive motility and a higher proportion of immotile sperm. Longer sexual abstinence duration was associated with higher sperm concentration. The proportion of explained variance was modest but consistent across analyses.

**CONCLUSION:**

These findings suggest that age and sexual abstinence duration are associated with selected semen parameters in normozoospermic men. The observed age-related differences in the associations with sexual abstinence and semen parameters warrant further investigation in larger, prospective studies before age-specific abstinence recommendations can be considered.

## INTRODUCTION

Infertility affects approximately 10–20% of couples worldwide, with male factors contributing to nearly half of cases^1^. Semen analysis remains the cornerstone of male fertility evaluation and is standardized according to World Health Organization (WHO) guidelines^2^.

Spermatozoa are produced in the seminiferous tubules and mature in the epididymis, where they acquire motility and fertilizing capacity^3,4^. This maturation process, together with ejaculation frequency, contributes to variability in semen parameters^4^. Accordingly, the WHO recommends 2–7 days of sexual abstinence before semen analysis to ensure standardization^2^. Despite this recommendation, the optimal sexual abstinence remains debated^4-7^. While WHO guidelines aim to standardize diagnostic evaluation, reproductive societies often recommend shorter intervals to optimize outcomes in assisted reproduction^8^. In parallel, delayed fatherhood has become increasingly common, raising interest in the effects of paternal age on semen quality. Although age-related changes in semen parameters have been reported, findings remain inconsistent^5^. Moreover, it is unclear whether sexual abstinence has similar effects across different age groups.

Therefore, this study aimed to investigate the associations of age and sexual abstinence with conventional semen parameters in normozoospermic men, with particular focus on whether these associations varied across age groups.

## METHODS

### Study design and population

This retrospective observational study was conducted at the Andrology Laboratory of Sivas Numune Hospital. Medical records of men who underwent semen analysis between May 2023 and February 2024 were reviewed. Men aged ≥18 years with complete semen data were included. Patients with prior varicocelectomy, grade III varicocele, active fertility treatment, or incomplete data were excluded. Patients with grade III varicocele or a history of varicocelectomy were excluded to reduce potential heterogeneity related to clinically significant varicocele and previous surgical intervention. Additionally, individuals with semen parameters below WHO 2010 reference limits were excluded to restrict the cohort to normozoospermic men^2^.

### Semen collection and analysis

Samples were collected by masturbation after the reported period of sexual abstinence and analyzed according to WHO 2010 guidelines. Sexual abstinence duration was recorded in days according to the laboratory's routine recording system. The predefined abstinence categories were 1–3 and 4–6 days. No participant in the final dataset had a recorded abstinence duration of 1 or 7 days; therefore, the observed abstinence durations ranged from 2 to 6 days.

### Statistical analyses

IBM Statistical Package for the Social Sciences (SPSS) Statistics version 20 (Chicago, IL, USA) was used for statistical analyses, and p<0.05 was the threshold for statistical significance. The Shapiro-Wilk test was used to examine the normality of the continuous data. Since the data did not exhibit normal distribution, the Mann-Whitney U test was used to compare semen parameters among the age and sexual abstinence groups. Spearman's correlation coefficient was used to examine the relationships between semen parameters, age, and days of sexual abstinence. Multivariate linear regression analysis was used to examine the associations of age and sexual abstinence on semen parameters. Exact two-sided p-values were reported for Mann-Whitney U tests

## RESULTS

The distribution of semen parameters according to age group is presented in Table 1. Mean semen volume decreased across age groups, whereas mean sperm concentration increased. Total and progressive motility showed lower mean values with advancing age, while non-progressive and immotile sperm proportions tended to increase.

**Table 1:** Distribution of semen parameters according to age group.

Semen parameter	20–29 years (n=228) mean (95%CI)	30–39 years (n=157) mean (95%CI)	40–49 years (n=27) mean (95%CI)
Semen volume (mL)	3.5 (3.3–3.7)	3.2 (3.1–3.5)	2.9 (2.5–3.3)
Sperm concentration (million/mL)	64.7 (59.9–69.5)	69.6 (62.6–76.6)	85.4 (66.2–104.6)
Total motility (%)	67.0 (65.5–68.5)	66.3 (64.6–68.0)	61.5 (57.0–66.0)
Progressive motility (%)	55.7 (54.1–57.3)	54.1 (52.1–56.0)	48.7 (44.3–53.1)
Non-progressive motility (%)	11.4 (10.8–12.1)	12.4 (11.6–13.3)	12.9 (10.6–15.1)
Immotile sperm (%)	33.1 (31.6–34.7)	34.0 (32.3–35.7)	38.8 (34.3–43.3)

CI: confidence interval.

After adjustment for sexual abstinence duration, each 1-year increase in age was associated with a 0.024 mL decrease in semen volume across the study population. In contrast, sperm concentration and the proportion of immotile sperm increased by 0.703×10^6^/mL and 0.203%, respectively, per year of age. Total motility and progressive motility decreased by 0.191 and 0.240%, respectively, with each additional year of age. When adjusting for age, each additional day of sexual abstinence was associated with an increase of 5.929×10^6^/mL in sperm concentration (Table 2).

**Table 2:** Multivariate linear regression analysis of age and sexual abstinence duration in relation to semen parameters.

		B	SE	β	95%CI	p	Model R^2^
Semen volume (mL)	Age	-0.024	0.011	-0.109	-0.046 to -0.003	**0.027**	0.018
	Sexual abstinence	0.151	0.089	0.083	-0.024 to 0.325	0.091	
Sperm concentration (10^6^/mL)	Age	0.703	0.327	0.105	0.060 to 1.347	**0.032**	0.024
	Sexual abstinence	5.929	2.640	0.110	0.739 to 11.120	**0.025**	
Total motility (%)	Age	-0.191	0.091	-0.103	-0.369 to -0.012	**0.036**	0.013
	Sexual abstinence	-0.605	0.732	-0.041	-2.043 to 0.833	0.409	
Progressive motility (%)	Age	-0.238	0.099	-0.118	-0.433 to -0.043	**0.017**	0.018
	Sexual abstinence	-0.959	0.803	-0.059	-2.532 to 0.626	0.233	
Non-progressive motility (%)	Age	0.053	0.042	0.062	-0.030 to 0.136	0.210	0.007
	Sexual abstinence	0.384	0.341	0.056	-0.285 to 1.054	0.260	
Immotile sperm (%)	Age	0.203	0.091	0.109	0.024 to 0.382	**0.027**	0.013
	Sexual abstinence	0.532	0.736	0.035	-0.915 to 1.978	0.471	

B: unstandardized regression coefficient; SE: standard error; β: standardized regression coefficient; 95%CI: 95% confidence interval; R^2^: coefficient of determination. Bold values indicate statistical significance (p<0.05).

In age-stratified analyses, a significant difference in semen volume was observed among men aged 20–29 years according to sexual abstinence. Semen volume was higher in individuals who abstained for 4–6 days compared with those who abstained for 1–3 days (p=0.01). Among men aged 30–39 years, sperm concentration was significantly higher in those with an abstinence period of 4–6 days compared with shorter sexual abstinence (p=0.03) (Table 3).

**Table 3:** Comparison of semen parameters according to sexual abstinence duration within age groups.

Age group	Semen parameter	1–3 days, mean (95%CI)	4–6 days, mean (95%CI)	Mann-Whitney U	Exact p-value
		n=120	n=108		
20–29 years (n=228)	Semen volume (mL)	3.32 (3.07–3.56)	3.79 (3.50–4.08)	5,191.0	0.009
	Sperm concentration (×10^6^/mL)	62.39 (55.52–69.26)	67.25 (60.50–74.01)	5,878.0	0.227
	Total motility (%)	66.89 (64.81–68.97)	67.07 (64.89–69.26)	6,399.0	0.871
	Progressive motility (%)	55.97 (53.72–58.21)	55.34 (53.04–57.64)	6,312.5	0.737
	Non-progressive motility (%)	11.02 (10.03–12.00)	11.90 (10.96–12.84)	5,909.5	0.251
	Immotile sperm (%)	33.38 (31.28–35.48)	32.87 (30.63–35.11)	6,302.5	0.722
30–39 years (n=157)		**n=70**	**n=87**		
	Semen volume (mL)	3.34 (3.02–3.67)	3.26 (3.00–3.53)	2,968.5	0.785
	Sperm concentration (×10^6^/mL)	60.32 (51.44–69.20)	77.05 (66.74–87.36)	2,438.5	0.032
	Total motility (%)	67.47 (64.80–70.15)	65.36 (63.13–67.59)	2,712.5	0.241
	Progressive motility (%)	55.41 (52.22–58.61)	52.97 (50.43–55.50)	2,730.5	0.268
	Non-progressive motility (%)	12.23 (10.85–13.61)	12.62 (11.65–13.60)	2,901.5	0.613
	Immotile sperm (%)	32.83 (30.18–35.48)	35.00 (32.79–37.21)	2,713.0	0.242
40–49 years (n=27) (exploratory)		**n=14**	**n=13**		
	Semen volume (mL)	3.07 (2.48–3.66)	2.69 (2.11–3.28)	70.5	0.312
	Sperm concentration (×10^6^/mL)	84.64 (61.44–107.84)	86.31 (51.31–121.31)	90.5	0.991
	Total motility (%)	62.50 (55.30–69.70)	60.38 (53.99–66.78)	82.0	0.676
	Progressive motility (%)	48.50 (41.55–55.45)	48.92 (42.66–55.19)	90.0	0.971
	Non-progressive motility (%)	14.21 (10.53–17.90)	11.46 (8.63–14.29)	63.5	0.187
	Immotile sperm (%)	37.79 (30.57–45.00)	39.85 (33.54–46.15)	83.0	0.711

Data are presented as mean (95%CI). P-values were calculated using the Mann-Whitney U test with exact two-sided significance values. Bold values indicate statistical significance (p<0.05). CI: confidence interval.

Correlation analyses demonstrated negative linear correlations between age and semen volume, total motility, and progressive motility, as well as a positive linear correlation between age and the proportion of immotile sperm (Figure 1). In addition, a positive linear correlation was observed between sexual abstinence and sperm concentration, indicating higher sperm concentrations with longer periods of abstinence (Table 4).

**Figure 1:**
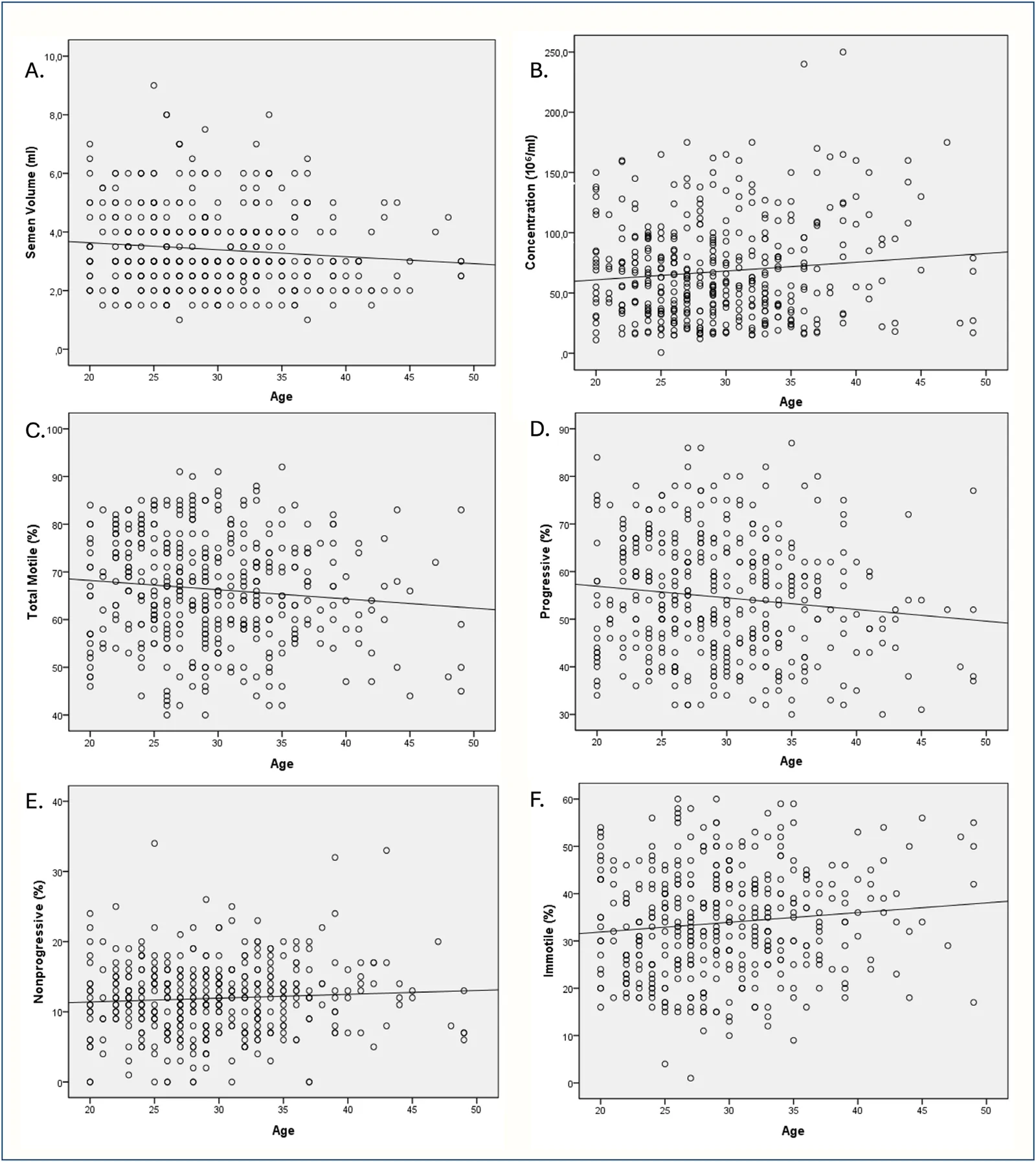
Scatterplots illustrating the unadjusted relationships between age and semen parameters. (A) Semen volume, (B) sperm concentration, (C) total motility, (D) progressive motility, (E) non-progressive motility, and (F) immotile sperm.

**Table 4:** Spearman correlations between age, sexual abstinence duration, and semen parameters.

	Age		Sexual abstinence	
	r_s_	p	r_s_	p
Semen volume (mL)	-0.097	**0.04**	0.086	0.08
Concentration (10^6^/mL)	0.052	0.29	0.109	**0.02**
Total motile (%)	-0.101	**0.04**	-0.041	0.40
-Progressive motility (%)	-0.113	**0.02**	-0.059	0.23
-Non-progressive motility (%)	0.049	0.31	0.049	0.32
Immotile sperm (%)	0.105	**0.03**	0.034	0.48

r_s_: Spearman's rank correlation coefficient; p: two-sided p-value. Bold values indicate statistical significance (p<0.05).

## DISCUSSION

Male reproductive function undergoes gradual changes with advancing age. Although male fertility can be preserved throughout life, age-related alterations in androgen production, spermatogenesis, and sexual function have been well documented. Increasing evidence suggests that advanced paternal age is associated with changes in semen quality, fertility potential, pregnancy outcomes, and offspring health^9^. Because age-related differences in sexual activity may also contribute to reproductive outcomes^10^, sexual abstinence represents a potentially relevant behavioral factor when interpreting age-related differences in semen parameters. The optimal duration of sexual abstinence remains controversial. Current guidelines differ in their recommendations, with WHO suggesting 2–7 days for standard semen analysis, whereas European Society of Human Reproduction and Embryology (ESHRE) recommends shorter intervals (3–4 days) in the assisted reproduction^2,8^. These recommendations are largely based on different clinical endpoints and therefore may not be directly interchangeable. Previous studies have reported differences in reproductive outcomes according to abstinence duration, including possible associations between shorter abstinence intervals and fertilization or pregnancy outcomes in assisted reproduction^11^. However, these findings involve different clinical endpoints from those evaluated in routine semen analysis. Therefore, the present study sought to explore whether the association between sexual abstinence and conventional semen parameters varied across age groups within a normozoospermic population.

In the present study, we evaluated semen volume, concentration, and motility in a strictly normozoospermic population, focusing on the associations of age and sexual abstinence with conventional semen parameters. We observed a modest decline in semen volume with increasing age, consistent with previous reports^12,13^. This finding has been attributed to age-related functional changes in the seminal vesicles, which contribute substantially to ejaculate volume and fructose secretion^5^. However, conflicting evidence exist, reporting either no association^14^ or even an increase in semen volume with age^15^, suggesting potential confounding factors.

Longer abstinence periods were associated with higher semen volume in men in their twenties. This observation may reflect enhanced secretory activity of the prostate and seminal vesicles following androgen stimulation^16^. Age-related differences in androgen-dependent accessory gland function may modulate the effect of sexual abstinence on semen volume, potentially making this relationship more apparent in younger men. Further studies are required to confirm this age-dependent effect.

In age-stratified analyses, longer abstinence was significantly associated with higher semen volume among men aged 20–29 years, whereas among men aged 30–39 years, longer abstinence was significantly associated with higher sperm concentration. No significant associations were observed for the other semen parameters within these age groups. The age-stratified findings provide preliminary evidence of potential age-dependent associations between sexual abstinence and semen parameters. Further studies with larger and more balanced age-stratified populations are needed to confirm the reproducibility and clinical relevance of these findings.

Sperm concentration increased with advancing age in our cohort, consistent with some previous studies^12,17^, although contradictory findings have also been reported^14,18^. Given the concurrent decline in semen volume with age, the observed increase in sperm concentration may partly reflect a concentration effect rather than increased spermatogenic output. Regarding sexual abstinence, we observed a positive association between longer abstinence and higher sperm concentration, consistent with previous evidence demonstrating a positive association between abstinence duration and sperm concentration^19,20^. Recent large-scale evidence has similarly demonstrated an increase in sperm concentration with longer abstinence among normozoospermic men, while no significant association with motility was observed^21^.

The selection of a normozoospermic population was intentional, as it allowed the associations between age, sexual abstinence, and conventional semen parameters to be evaluated within a more homogeneous semen profile. However, this design may have limited the range of semen parameter values and consequently reduced the ability to detect stronger associations. Recent evidence further supports the relevance of studying age-related changes even among normozoospermic men. In a 2026 study of 534 normozoospermic men, advancing paternal age was associated with lower ejaculatory volume and progressive motility, as well as higher sperm DNA fragmentation, indicating that age-related alterations may be detectable even when conventional semen parameters remain within the normal range^22^. These findings support our rationale for examining age-related associations within a normozoospermic population rather than restricting the analysis to men with overt semen abnormalities.

In the multivariable analysis, each additional day of sexual abstinence was associated with an increase in sperm concentration, although the relatively wide 95%CI indicates uncertainty around the magnitude of this association. This finding should therefore be interpreted as a modest population-level association rather than as a basis for predicting an individual patient's sperm concentration.

Total and progressive sperm motility declined with increasing age, in agreement with the majority of published data^6^. These changes may be related to age-associated dysfunction of post-testicular organs, particularly the epididymis, where sperm acquire motility^23^. Age-related reductions in prostate- and epididymis-derived secretory markers, as well as increased oxidative stress, may contribute to impaired sperm motility^24^.

The relationship between sexual abstinence and sperm motility remains controversial. While several studies report reduced motility with prolonged abstinence, these findings often involve oligospermic or asthenozoospermic populations^25^. A recent study of 3,052 men undergoing fertility evaluation similarly found that longer abstinence was associated with higher sperm concentration but lower progressive and total motility^26^. In our normozoospermic cohort, sexual abstinence duration was not significantly associated with total, progressive, non-progressive, or immotile sperm proportions in the overall multivariable analysis. Furthermore, the age-stratified analyses did not demonstrate significant differences in motility-related parameters according to abstinence duration.

In accordance with the study protocol, individuals with semen parameters below the WHO 2010 lower reference limits (<5th percentile) were excluded, resulting in a cohort restricted to normozoospermic men. While this approach improved cohort homogeneity, it may also have introduced range restriction by reducing variability in semen parameters and potentially attenuating the magnitude of detectable associations. Accordingly, the present findings should be interpreted within the context of a normozoospermic population and should not be directly generalized to men with abnormal semen parameters.

In addition, several limitations should be considered. Sperm morphology was not included, because it was not recorded in a sufficiently standardized and consistently available manner in the retrospective dataset; therefore, a fully comprehensive assessment of semen quality was not possible. The relatively small proportion of older individuals, particularly the 40–49-year age group (n=27), may limit the precision and generalizability of the age-stratified findings, and results for this subgroup should therefore be considered exploratory. Furthermore, the retrospective design limited our ability to adjust for potential confounding factors, including BMI, smoking, alcohol consumption, comorbidities, and medication use, and residual confounding cannot be excluded. The single-center design may also limit the generalizability of the findings to other populations and geographic settings, as semen parameters may vary across populations. The relatively low R^2^ values across the regression models represent an additional limitation, as age and sexual abstinence duration accounted for only a limited proportion of the variability in the examined semen parameters. This finding is consistent with the multifactorial nature of semen characteristics and the limited number of covariates available in our retrospective dataset. Accordingly, the statistically significant associations observed should be interpreted as modest population-level associations rather than individually predictive effects or as a basis for clinical thresholds or age-specific abstinence recommendations.

At the same time, the study has several strengths, including the use of standardized WHO 2010 criteria, single-laboratory analysis, and multivariate modeling, allowing the associations of age and sexual abstinence to be evaluated simultaneously. Age was associated with several semen parameters, particularly motility-related parameters, whereas sexual abstinence showed its most consistent association with sperm concentration.

Our findings suggest that the associations between sexual abstinence duration and conventional semen parameters may vary across age groups in normozoospermic men. These age-dependent findings should be considered exploratory and hypothesis-generating rather than as a basis for age-specific abstinence recommendations, and warrant confirmation in larger prospective and multicenter studies.

## CONCLUSION

Advancing age was associated with measurable changes in semen quality in normozoospermic men. Longer sexual abstinence duration was associated with higher sperm concentration, while age-stratified analyses suggested that the relationship between abstinence duration and selected semen parameters may vary across age groups. These findings highlight the importance of considering age when interpreting semen analysis and warrant further investigation in larger, prospective studies to determine whether age-related differences in sexual abstinence duration have clinically meaningful implications.

## Data Availability

The datasets generated and/or analyzed during the current study are available from the corresponding author upon reasonable request.
